# How to leverage medication use evaluations for antimicrobial stewardship goals: a primer for physicians

**DOI:** 10.1017/ash.2025.10189

**Published:** 2025-10-13

**Authors:** Ritika Prasad, Radhika Arya, Lina Meng, Marisa Holubar, William Alegria, Alex N. Zimmet

**Affiliations:** 1 Division of Infectious Diseases and Geographic Medicine, Stanford University School of Medicine, Stanford, CA, USA; 2 Stanford Antimicrobial Safety and Sustainability Program, Stanford Health Care, Stanford, CA, USA

## Abstract

Medication Use Evaluations (MUEs) are structured quality improvement tools used to optimize medication use within healthcare systems. MUEs can explore safety concerns, high costs, or inappropriate use associated with any medication, among other factors. This can offer a valuable opportunity for Antimicrobial Stewardship Programs (ASPs) to promote stewardship goals, which often overlap with these concerns for specific antimicrobials. MUEs can also provide an avenue to promote interdisciplinary collaboration with targeted groups relevant to the ASP’s goals as well as an opportunity to give trainees ownership of system-facing stewardship projects. Stewardship pharmacists may often have experience with leading this process as part of their training, but MUEs represent a gap in training for physicians. Here we provide guidance on how stewardship physicians can engage in the MUE process, from identifying relevant topics to interpreting findings and supporting implementation efforts, allowing them to contribute effectively even without prior MUE experience.

You are a new ASP physician attending a Pharmacy and Therapeutics meeting. You are asked by a pharmacist for input on designing a medication use evaluation for cytomegalovirus immune globulin, which may be widely misused. But first, you find yourself thinking, “Wait, what’s an MUE?”

## Introduction

Medication Use Evaluations (MUEs) are quality improvement processes designed to assess and optimize appropriate, safe, and cost-effective use of medications within a healthcare system. Similar terms, such as Drug Utilization Evaluations (DUEs), Drug Utilization Reviews (DURs), Medication Use Management, and Pharmaceutical Use Management, are often used with minor distinctions. Originating in the 1960s in the United Kingdom and Northern Europe as part of broader quality assurance efforts,^1^MUEs have since gained momentum worldwide. In the United States, the Joint Commission mandates that hospitals establish formal processes for monitoring and evaluating medication use^2^ and MUEs are often used as a systematic method to accomplish this goal.

MUEs can investigate various areas related to medication use, including medication safety, formulary compliance, cost-effectiveness, or patterns of use that could affect patient outcomes. They are typically reviewed and implemented by the Pharmacy and Therapeutics Committee, an entity responsible for formulary management and optimizing medication use within a health system. Input from key stakeholders, such as prescribers and pharmacists frequently involved in the medication use process, is essential to ensure successful implementation and practice change.

While MUEs are commonplace to pharmacy departments, you, as an antimicrobial stewardship program (ASP) physician, may be asked to contribute to an MUE without prior experience or exposure to the process. In this primer, we aim to bridge that gap by outlining the value of MUEs for ASP and highlighting how you can effectively contribute to MUEs to advance stewardship objectives.

## Leveraging MUEs for ASP

MUEs are a valuable tool in your ASP arsenal. The CDC highlights MUEs as a useful mechanism to track antimicrobial use and measure the impact of ASP interventions.^3^ An MUE focused on antimicrobial use typically includes the following key components: clearly defined objectives, background and rationale for the evaluation, specific criteria for assessing appropriateness, methods for data collection and analysis, results with interpretation, and actionable conclusions and recommendations.^4^ This approach can be a more formal, structured, and rigorous process than other ASP workflows, and can allow you to investigate patterns of suboptimal antimicrobial use or other issues related to antimicrobials, identify areas for targeted interventions, and inform development of restriction criteria, formulary updates and institutional guidelines.

**Figure 1.**
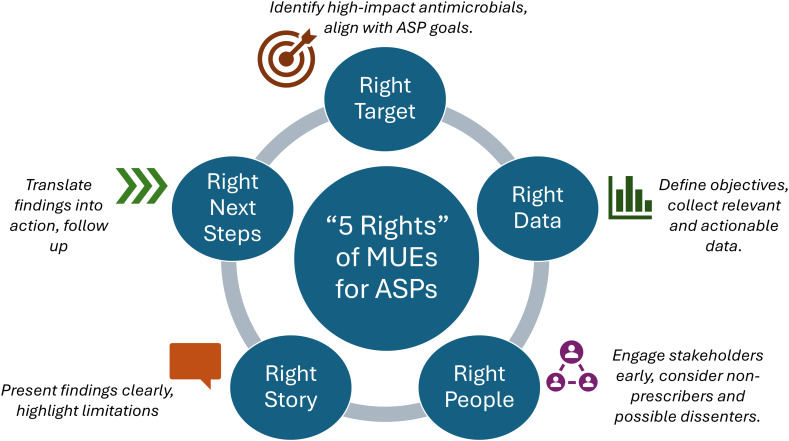
The “5 rights” of medication use evaluations for antimicrobial stewardship programs. MUE, medication use evaluation; ASP, antimicrobial stewardship program.

To craft a successful MUE, we propose a framework of five foundational elements: identify the right target, get the right data, engage the right people, tell the right story, and plan the right next steps (Figure 1). With this method, you can ensure that the MUE is aligned with antimicrobial stewardship goals and positioned to translate findings into action while avoiding common pitfalls (Table 1).

**Table 1.** Common pitfalls and pearls for medication use evaluations in antimicrobial stewardship

MUE component	Common pitfall	Pearls
Target	Choosing a drug without clear rationale or impact	Ask: “Why is this MUE needed?” and “What impact do I want it to have?” Tailor to your program’s setting and priorities.
Data	Collecting data without a defined objective or criteria for “appropriate” use; drawing strong conclusions about clinical outcomes	Refine objectives first, define patient population and appropriateness criteria early.
People	Not engaging teams who drive management decisions or those who may resist recommended changes	Identify clinical owners and potential dissenters early. Emphasize shared goals and collaborative intent when reaching out.
Story	Presenting results without clearly stating conclusions, limitations, or practical implications	Use clear messaging, highlight limitations, and proactively seek feedback.
Next Steps	Lacking a plan for rollout, feedback, or follow-up	Plan communication strategy and monitoring. Consider publicizing at unit-based quality meetings and using the PDSA cycle to gather follow-up data.

MUE, medication use evaluation.

## Identify the right target

Selecting the right target is the critical first step in conducting an MUE. Before committing time and resources, you should ask, “Why is this MUE needed?” and “What kind of impact do I want the MUE to have?”The focus of an MUE can vary widely depending on your priorities; inappropriately focused MUEs are a common pitfall that may lead to extensive effort with little stewardship fruit at the end. Occasionally the medications of interest to other groups (*eg,* a Pharmacy and Therapeutics Committee) may not be those you’re most interested in—this challenge can be an opportunity for dialog to identify other overlapping interests and ensure you evaluate the right medication for all involved. Consider the following when identifying potential targets:

- Antimicrobials with high utilization: By assessing commonly used antimicrobials, you can gain further insight on clinically relevant, high-impact targets. For example, one MUE of levofloxacin found high rates of inappropriate use primarily due to lack of microbiologic data and incorrect indications.^5^ This data can then be used to implement restriction criteria or update institutional guidelines.
- New antimicrobials added to formulary: Conducting an MUE shortly after a new antimicrobial is introduced to market can help your institution rapidly assess appropriate use, develop restriction criteria targeting the right patient population, and monitor adverse drug reactions.
- Dosing and adverse events: Prospective MUEs investigating dosing errors and adverse events can significantly improve antimicrobial prescribing practices and patient outcomes.^6^ Internal metrics such as incident reports or stewardship case reviews may help you prioritize which antimicrobials to evaluate.
- Antimicrobials with cost considerations: High cost can significantly influence optimal prescribing, but actual costs are often not clear to frontline providers. MUEs can help clarify this. For example, we reviewed patients discharged on fidaxomicin and found that approximately 80% of patients had low copayments, and only 27% required prior authorization, suggesting that the economic barriers to fidaxomicin were less of a barrier than believed at the time.^7^
- Antimicrobials with low evidence or variability in clinical practice: Consider leveraging MUEs to address variability in clinical practice, which often occurs when prescribing decisions lack robust guideline support. Niche antimicrobials with limited evidence-based guidance, such as cytomegalovirus immune globulin (CMV-IGIV),^8,9^ may be ideal candidates.
- Operational Red Flags: FDA warnings, increased resistance, or documented clinical failures should serve as cues to initiate a timely MUE so that you can proactively identify and mitigate these risks. Additionally, the Pharmacy and Therapeutics Committee may request a MUE after a change in restriction criteria or formulary change to ensure limited harm.
- Impact of ASP interventions: Although MUEs are not designed to be clinical trials measuring patient outcomes (see “Get the Right Data” below), measuring pre and postintervention process outcomes can be a helpful exploratory exercise to gauge the impact of an ASP intervention. For example, one MUE demonstrated that a pharmacist-led educational intervention significantly reduced inappropriate use of piperacillin/tazobactam from 31% to 12%, and improved multiple quality indicators including renal dose adjustment, timely bacterial culture orders, de-escalation, and adherence to extended-infusion protocols.^10^

## Get the right data

Once you’ve identified your target, start refining the objective of the MUE and your desired outcome. Most MUEs require at least some chart review, and you can maximize efficiency by outlining what you’re looking for before you start. For example, if your objective is to assess and improve adherence to established restriction criteria for an antimicrobial, you will want details about the non-adherent prescribing—both the clinical situations and the gaps in the restriction process. If your objective is to assess current use and develop restriction criteria, you will need to identify common prescribing themes to inform your decisions. Understanding which prescribers are using the targeted antimicrobial and how they are using it (eg, specific patient populations or conditions) will allow you to identify non-evidence-based practices and streamline the restriction criteria development and implementation process. If your objective is to assess a new antimicrobial for formulary inclusion, consider whether your institution treats patient populations similar to those studied in the drug’s clinical trials to help determine clinical relevance and utility.^11^

A full list of potential data elements to include are discussed elsewhere.^11^ Some points to consider:

- Define the patient population: Give particular attention to how you define commonly used and heterogeneously defined clinical categories relevant to the MUE - like “immunocompromised” for CMV-IGIV.
- Establish a definition for appropriate use: How will you consistently assess if each use of the target antimicrobial was “appropriate” or not? Defining this early will streamline chart review. It’s often useful to trial your definition on a handful of cases to flesh out gray areas early and establish a way to categorize them. Commonly used sources to establish appropriate use include published guidelines (institutional or national), restriction criteria, and clinical trials used to establish FDA approval.
- Leverage your Electronic Medical Record (EMR): Are there data that can help you understand the ordering process or gain insight into the clinician’s rationale for antimicrobial use. For example, some EMRs have a mandatory clicked indication field for antibiotic orders.
- Assess for adverse events: Which are of interest? What details are relevant to describe potential harm and how will you categorize these events?
- Be wary of including clinical outcomes data: MUEs are designed to evaluate the use of an antimicrobial, not its treatment effect. Although systematic and often rigorously done, MUEs are not clinical studies. Among other considerations, patient populations are typically too small to draw any conclusions about clinical outcomes, selection bias is unavoidable, and many confounders remain unexamined. Emphasizing this point to the MUE’s audience is also important to avoid over- and mis-interpretation of its results.

## Engage the right people

MUEs require collaboration with interprofessional teams across your health system. This can be a powerful opportunity for new stewardship teams or members to build strong relationships with colleagues in other disciplines—both to establish trust and to identify opportunities for future joint stewardship activities. Start by including the group that most commonly drives management of the relevant medication or patient population—this is often the primary/admitting team (such as certain organ transplant teams for CMV-IGIV) or the infectious diseases consult team. Consider who holds local “ownership” of the drug or disease entity in question, as institutional culture often varies as to which team may drive management decisions. If a drug or relevant patient population is managed by multidisciplinary teams, you may need to engage representatives from each.

Seek also to include relevant non-physician stakeholders. Frontline pharmacists can be invaluable, both in accurately identifying MUE opportunities that fit within the stewardship mission as well as providing unique insight into the decision-making and practical processes that influence antimicrobial use. These pharmacists and their trainees are often well positioned to both identify and help execute MUEs themselves, and may even undertake MUEs as a dedicated stewardship experience. This provides an opportunity for you to integrate with pharmacy departments on the level of professional development. Bedside nurses are another group with unique perspectives, playing an integral role in implementing interventions.^14^ Involving these groups will strengthen execution and implementation of your MUE and give you a chance to educate multidisciplinary colleagues to spread a culture of stewardly practice throughout your health system.

Finally, consider any stakeholders who could disagree with the MUE’s conclusions. Listening to input from these individuals or groups and centering your shared goal of improving patient outcomes early in the process is essential. Even if the MUE does not “change hearts and minds”, by reaching out to these stakeholders, you can move the conversation in the right direction and plant the seed for eventual practice change.

## Tell the right story

After the data collection and analysis phase, the results of an MUE are typically presented and reviewed in a Pharmacy and Therapeutics Committee meeting (or another similar forum). This meeting commonly involves a “vote to approve” the MUE’s findings and recommendations. If you are presenting the MUE, the following tips can help you tell a compelling story to engage your audience and ensure they understand the main takeaways (and limitations) of the MUE.

- Identify the major conclusions: Use clear and concise messaging to communicate the findings of the MUE and contextualize how the data should be interpreted to inform future practice. For example, if you recommend a change in restriction criteria, explain why your data supports your recommendations. Remember to also highlight limitations of the data and discourage over-interpretation of results.
- Discuss outlier situations or cases: There may be scenarios where the conclusions don’t apply, or you may have data points that clearly deviate from the majority. Acknowledge these outliers, while guiding the group to practical, evidence-based recommendations that apply in most cases. If the data are too noisy to support unifying conclusions, you might highlight this as well.
- Ask for feedback: Use this opportunity to seek feedback on the MUE’s conclusions from committee members, who are often institutional leaders with substantial experience and wisdom.^13^ Ahead of the meeting, proactively ask for feedback from those who lead or have significant influence on the group to secure “buy-in” and anticipate any potential roadblocks.

## Plan the right next steps

After the MUE’s findings are reviewed and recommendations approved, the next step is to translate these decisions into action. This can be an additional barrier to a successful MUE, but one you can address with some preemptive planning. Although your strategy will depend on your MUE’s unique goals, consider some of these common strategies and their challenges:

- Plan your communication campaign:Pair actionable changes with communication to affected providers. Email announcements and educational outreach can reach frontline providers, and hospital unit-based quality meetings can help engage clinical and administrative leaders. Keep in mind that your ASP-oriented perspective may not be shared by all prescribers; these leaders may be able to help facilitate challenging conversations around sensitive topics.
- Anticipate the nuances of updating restriction criteria: If you recommend restriction criteria to encourage appropriate use, consider who is responsible for approval—the stewardship team or the order verification pharmacist? Consider the frequency and likelihood of suboptimal use when deciding. If there is a high likelihood of misuse, particularly for novel or extremely broad-spectrum agents, consider ID consultation as a condition for approval if resources permit.
- Design effective clinical guidelines: This approach may be best suited for antimicrobials with very specific uses, such as fidaxomicin. In the example of CMV-IGIV, specific transplant protocols would be a similar opportunity to formalize recommendations for use. Antimicrobials used for many indications, such as anti-pseudomonal beta-lactams, may be more challenging to steward in this way. A more focused approach, such as guidelines targeting common conditions with high prevalence of inappropriate use, like community-acquired pneumonia—could have more meaningful impact.
- Adopt the PDSA (Plan-Do-Study-Act) cycle: Consider using the PDSA cycle, a widely used iterative quality improvement methodology that has been well-described. For example, it may be helpful to monitor postMUE stewardship metrics to assess appropriateness or compliance with restriction criteria. These metrics can inform the need for additional interventions and could also serve as the basis for follow-up MUEs.

## Conclusion

MUEs are a powerful tool for ASPs, enabling data-driven decision-making to guide the development of recommendations and interventions. MUEs allow ASPs to advance stewardship goals through a structured, rigorous and action-oriented approach. MUEs also offer the added benefit of enhancing the institutional visibility of ASP initiatives. Importantly, the process fosters collaboration by bringing together key stakeholders from different disciplines across an institution to align shared priorities. For you as an ASP physician, gaining experience with MUEs offers several professional advantages, including a deeper understanding of institutional quality improvement pathways, opportunities for demonstrating leadership, and potential avenues for scholarly publication. Ultimately, MUEs not only support the core mission of antimicrobial stewardship but also serve as a strategic platform for institutional engagement, interdisciplinary collaboration, and professional development for ASP physicians.
